# Autoimmune diseases and diffuse large B-cell lymphoma: A Mendelian randomization study

**DOI:** 10.1097/MD.0000000000042855

**Published:** 2025-06-20

**Authors:** Jiaying Duan, Litian Ma, Tianhao Wang, Tian Li, Yu Li

**Affiliations:** a Department of Oncology, First Affiliated Hospital, Heilongjiang University of Chinese Medicine, Harbin, Heilongjiang Province, China; b Department of Thoracic Surgery, Tangdu Hospital, Air Force Medical University (The Fourth Military Medical University), Xi’an, China; c Internal Medicine of Traditional Chinese Medicine, The First School of Clinical Medicine, Heilongjiang University of Chinese Medicine, Haerbin, Heilongjiang Province, China; d Tianjin Key Laboratory of Acute Abdomen Disease-Associated Organ Injury and ITCWM Repair, Institute of Integrative Medicine of Acute Abdominal Diseases, Tianjin Nankai Hospital, Tianjin Medical University, Tianjin, China.

**Keywords:** autoimmune diseases, diffuse large B-cell lymphoma, genome-wide association study, Mendelian randomization

## Abstract

The causal link between autoimmune diseases (ADs) and diffuse large B-cell lymphoma (DLBCL) remains uncertain. This study aims to assess the causal effects of ADs on DLBCL risk using Mendelian randomization (MR). The summary dataset for ADs and lymphoma genome-wide association study (GWAS) was sourced from the open GWAS website. Single nucleotide polymorphisms were chosen as genetic instrumental variants based on linkage disequilibrium with *P* < 5 × 10^−8^ and *R*^2^ = 0.01 in different ADs GWAS. Palindrome and outlier single nucleotide polymorphisms were excluded. Cochran Q test, the MR-EGGER intercept test, MR-PRESSO, and leave-one-out analysis were used to assess sensitivity. Our results showed genetic liability to 6 ADs, including mixed connective tissue disease (odds ratios, OR_WM_1.578; 95% confidence intervals [CI]: 1.250–1.991, *P* < .001), psoriasis (OR_MR-Egger_ = 0.775; 95% CI: 0.604–0.992, *P* = .049), Sjögren syndrome (OR_IVW_ = 1.290; 95% CI: 1.072–1.551, *P* = .007), systemic lupus erythematosus (OR_IVW_ = 1.153; 95% CI: 1.053–1.262, *P* = .002), type 1 diabetes mellitus (OR_IVW_ = 0.899; 95% CI: 0.862–0.938, *P* < .001), and ulcerative colitis (OR_MR-Egger_ = 1.648; 95% CI: 1.210–2.243, *P* = .003) may have a causal relationship with DLBCL. Our MR results showed that ADs, such as Sjögren syndrome and systemic lupus erythematosus, may have causal relationship with DLBCL, while type 1 diabetes mellitus could reduce the risk of DLBCL.

## 1. Introduction

Neoplasms remain the main killer worldwide.^[1–6]^ Among which, B-cell lymphomas are a diverse set of malignant disorders affecting B lymphocytes, with variations in cause, clinical symptoms, and tissue structure. Immune deficiency and autoimmune conditions are regarded as significant risk factors for B-cell lymphomas.^[7]^ Epidemiological studies over the past 20 years have discovered connections between different BCL subtypes and numerous risk factors.^[8,9]^ Non-Hodgkin lymphomas (NHLs) primarily originate from B-cells, making up 85% to 90% of all NHL cases, with diffuse large B-cell lymphoma (DLBCL) being the most prevalent subtype, comprising 25% to 45% of new lymphoma diagnoses annually.^[10]^ Autoimmune diseases (ADs) occur when the body attacks its own antigens, leading to damage of its tissues and organs. Among these are conditions like rheumatoid arthritis (RA), systemic lupus erythematosus (SLE), and ankylosing spondylitis (AS), among others.^[11]^ At present, the pathogenesis of ADs is still unclear in modern medicine, and there is no specific drug for treatment, which has become an international public health problem that cannot be ignored.

Numerous studies have shown that patients with ADs are at an elevated risk for certain cancers, with DLBCL being the most common subtype in this context. Recent studies have indicated that the incidence of DLBCL in patients with SLE is 2 to 3 times higher than in the general population.^[12]^ In addition, the association of Sjögren syndrome (SS) with different lymphoma subtypes is a well-recognized. Research indicates a potential connection between SS and the onset of DLBCL, with SS patients having a relative risk of developing DLBCL between 2.0 and 6.57.^[9,13]^ Studies conducted recently have examined the causal connection between DLBCL and inflammatory bowel disease (IBD) using Mendelian randomization (MR), proposing that IBD might be a risk factor for DLBCL.^[14]^ The connection between DLBCL and other widespread autoimmune disorders is, however, still underexplored.

This research focused on examining combined data from a genome-wide association study (GWAS) through MR to explore the causal impact of several common ADs on the likelihood of developing DLBCL.

## 2. Methods

### 2.1. Data sources for ADs and DLBCL GWAS

GWAS consortia data, which is publicly available, formed the foundation for the MR analysis. Each dataset, such as the UK Biobank, the FinnGen study, and other large consortia when available, was analyzed on its own. For the FinnGen study, the DF9 version of the GWAS database was used to ensure the reliability of the results. The GWAS database for DLBCL data was obtained from FinnGen, a resource that comprises genetic information from more than 500,000 individuals in Finnish biobanks, coupled with digital health records from the Care Register for Health Care, and details from cancer, cause of death, and medication records.^[15]^ The DLBCL GWAS data consisted of 1010 cases and 287,137 controls. All the GWAS datasets mentioned are accessible to the public and can be obtained from the OPEN GWAS website. Table S1, Supplemental Digital Content, https://links.lww.com/MD/P256 contains detailed information.

### 2.2. Selection of genetic instrumental variants (IVs)

Following the 3 fundamental principles of MR study design closely: (1) a robust link between IVs and exposure factors; (2) IVs are not influenced by confounders in the exposure–outcome relationship; and (3) genetic variation influences outcomes solely through exposure, without other mechanisms: single nucleotide polymorphisms (SNPs) that meet the genome-wide significance level (*P* < 5 × 10^−8^) were chosen as IVs from the ADs GWAS.^[16]^ The linkage disequilibrium of *r*^2^ = 0.01 and clumping distance = 10,000. SNPs were excluded if they were palindromic with intermediate allele frequencies during the harmonization of the ADs and diffuse large B-cell lymphoma GWAS. Furthermore, SNPs absent from the outcome GWAS were omitted, and proxy SNPs were not utilized in this research. The SNPs that remained were employed for MR analysis. The F statistics were computed to evaluate the robustness of genetic instrumental variables. SNPs with an F statistic >10 were deemed strong genetic instruments. Tables S2 to S12, Supplemental Digital Content, https://links.lww.com/MD/P256 contain detailed information about these IVs.

### 2.3. Statistical analysis

To evaluate the causal effects of ADs on DLBCL, 3 MR methods were applied: inverse variance weighted (IVW), MR-Egger, and weighted median (WM). The IVW method provides the most precise estimates when all genetic instruments are valid, while MR-Egger and WM are robust to horizontal pleiotropy and outliers, respectively. Odds ratios (ORs) and 95% confidence intervals (CIs) were calculated, with OR > 1 indicating a risk-increasing effect. The study utilized the open-source statistical software R (version 4.1.2; R Foundation for Statistical Computing) along with the R packages TwoSampleMR (version 0.5.6), MR-PRESSO (version 1.0), and MendelianRandomization (version 0.6.0), and data.table (version 1.14.2).

To address potential biases, we systematically evaluated pleiotropy and heterogeneity. Pleiotropy:

MR-Egger intercept test^[17]^: this test detects directional pleiotropy by examining the intercept term in MR-Egger regression. A significant intercept (*P* < .05) suggests SNPs with effects on DLBCL independent of ADs. MR-PRESSO: this method identifies and corrects outlier SNPs that may introduce pleiotropy.^[18]^ The Global Test assesses overall pleiotropy, while the Distortion Test ensures corrected estimates align with IVW results. Heterogeneity: Cochran Q test: evaluates between-SNP heterogeneity in effect estimates. Significant heterogeneity (*P* < .05) prompted the use of fixed-effects models for robustness. Leave-one-out analysis: removing each SNP individually validated the stability of results.

Ethical approval was waived because this study exclusively used publicly available, de-identified GWAS summary data and did not involve direct patient contact or new patient data collection. Thus, informed consent and institutional review board approval were not required.

## 3. Results

### 3.1. SNP selection and harmonization

Genome-wide significant (*P* < 5 × 10⁻⁸) and independent SNPs were extracted from various ADs GWAS. Palindromic SNPs were removed when the exposure and outcome GWAS were subsequently harmonized. Finally, the remaining genetic variants strongly associated to DLBCL in each ADs were used for follow-up MR analysis, and the genetic IVs selected for each ADs were described in supplementary materials (Tables S2–S12, Supplemental Digital Content, https://links.lww.com/MD/P256).

### 3.2. Data preprocessing and feature engineering

**Figure 1.**
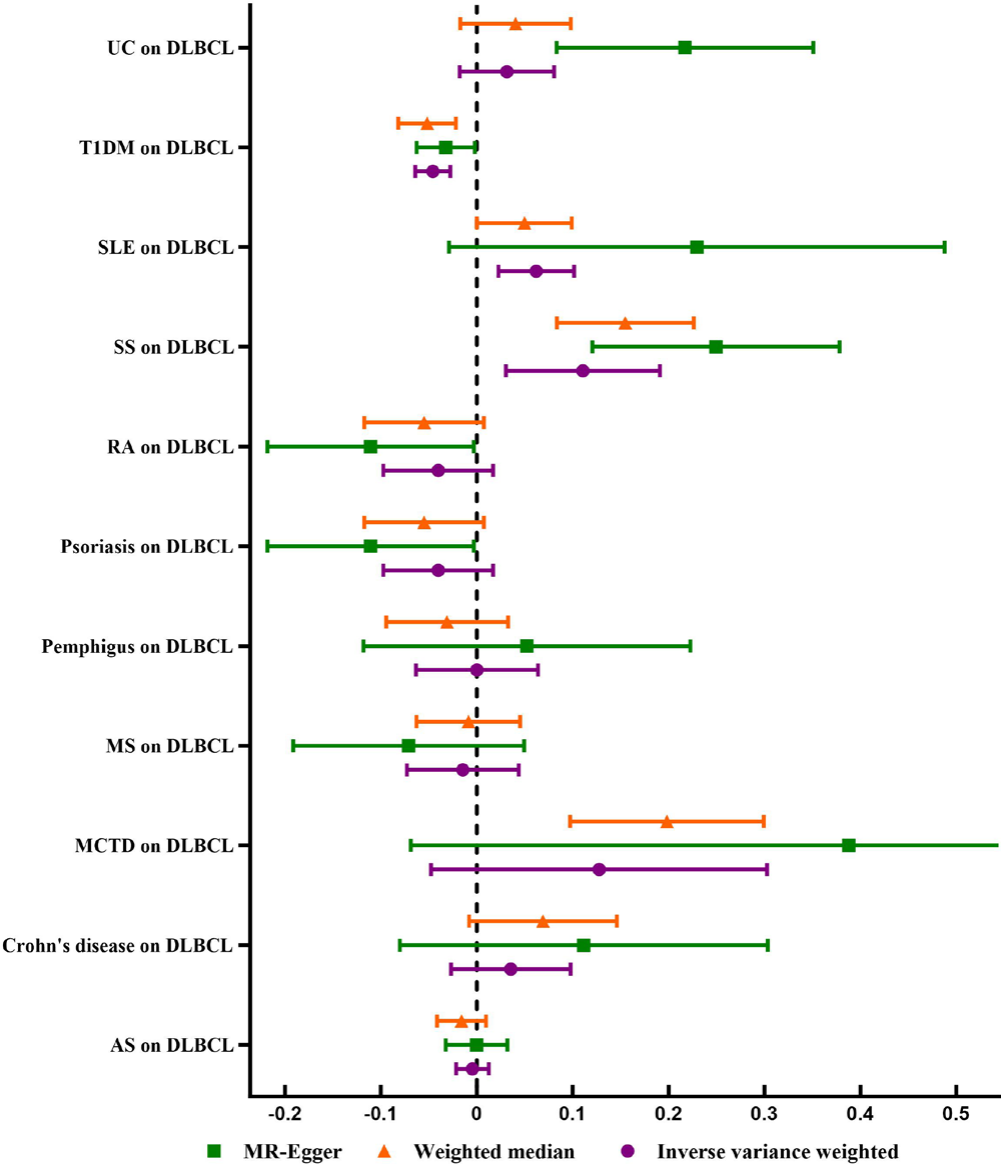
Causal association of 11 ADs with diffuse large B-cell lymphoma (DLBCL). ADs = autoimmune diseases.

**Figure 2.**
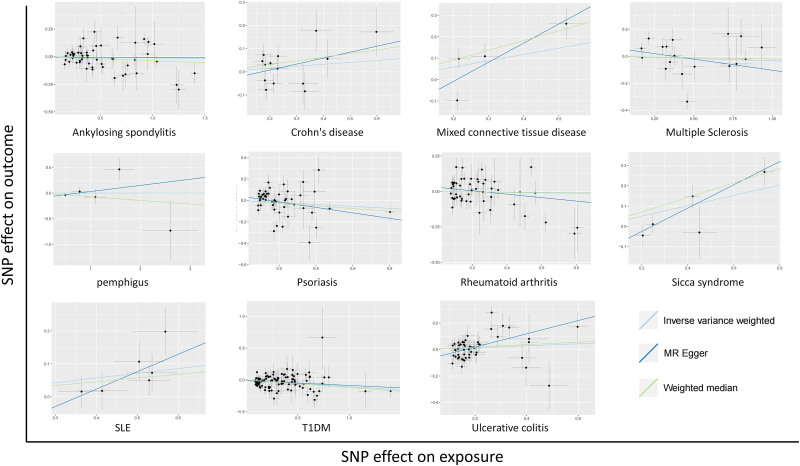
The scatter plot illustrates the connection between genetically predicted ADs and diffuse large B-cell lymphoma (DLBCL). ADs = autoimmune diseases.

The general causal estimates verified the causal relationship between ADs and DLBCL (Table 1), as shown in the forest plot (Fig. 1). As shown in Table 2, two-sample MR analysis indicated that genetically determined liability to 5 of the 11 studied ADs had no causal effect on DLBCL, including AS (OR_IVW_ = 0.989; 95% CI: 0.951–1.028, *P* = .593), Crohn disease (OR_IVW_ = 1.341; 95% CI: 0.896–2.007, *P* = .154), multiple sclerosis (MS, OR_IVW_ = 0.967; 95% CI: 0.845–1.105, *P* = .624), pemphigus (OR_IVW_ = 1.000; 95% CI: 0.863–1.157, *P* = .999), and RA (OR_IVW_ = 0.991; 95% CI: 0.892–1.099, *P* = .854). Genetically determined liability to the remaining 6 ADs such as mixed connective tissue disease (MTCD, OR_WM_ = 1.578; 95% CI: 1.250–1.991, *P* < .001), psoriasis (OR_MR-Egger_ = 0.775; 95% CI: 0.604–0.992, *P* = .049), SS (OR_IVW_ = 1.290; 95% CI: 1.072–1.551, *P* = .007), SLE (OR_IVW_ = 1.153; 95% CI: 1.053–1.262, *P* = .002), type 1 diabetes mellitus (T1DM, OR_IVW_ = 0.899; 95% CI: 0.862–0.938, *P* < .001), and ulcerative colitis (UC, OR_MR-Egger_ = 1.648; 95% CI: 1.210–2.243, *P* = .003) may have a causal relationship with DLBCL. Moreover, the causal effect estimates of SS (OR_MR-Egger_ = 1.766, 95% CI: 1.320–2.390, *P* = .032; OR_WM_ = 1.428, 95% CI 1.211–1.684, *P* < .001), SLE (OR_WM_ = 1.120; 95% CI: 1.000–1.256, *P* = .049), and T1DM (OR_MR-Egger_ = 0.927, 95% CI 0.865–0.995, *P* = .038; OR_WM_ = 0.887, 95% CI 0.828–0.950, *P* = .001) on DLBCL from the other MR models were consistent. The detailed information of the risk of DLBCL in patients with ADs was demonstrated in scatter plot (Fig. 2).

**Table 1:** Estimates of genetically liability to DLBCL on different immune diseases by two-sample Mendelian randomization.

Exposure_and_Outcome	MR model	Beta	SE	*P*val	OR
	IVW	-0.011	0.020	.593	0.989 (0.951–1.028)
AS on DLBCL	MR-Egger	-0.001	0.038	.977	0.998 (0.927–1.075)
	WM	-0.037	0.030	.219	0.963 (0.908–1.022)
Crohn disease on DLBCL	MR-Egger	0.257	0.225	.277	1.292 (0.831–2.010)
	WM	0.159	0.090	.079	1.172 (0.981–1.399)
	IVW	0.294	0.206	.154	1.341 (0.896–2.007)
MCTD on DLBCL	MR-Egger	0.893	0.537	.238	2.443 (0.853–6.995)
	WM	0.456	0.119	**<.001**	1.578 (1.250–1.991)
	IVW	-0.034	0.068	.624	0.967 (0.845–1.105)
MS on DLBCL	MR-Egger	-0.164	0.141	.263	0.849 (0.643–1.120)
	WM	-0.020	0.063	.747	0.979 (0.865–1.109)
	IVW	<0.001	0.075	.999	1.000 (0.863–1.157)
Pemphigus on DLBCL	MR-Egger	0.120	0.200	.591	1.128 (0.761–1.670)
	WM	-0.072	0.075	.336	0.931 (0.804–1.077)
	IVW	-0.093	0.067	.168	0.911 (0.798–1.039)
Psoriasis on DLBCL	MR-Egger	-0.255	0.127	**.049**	0.775 (0.604–0.992)
	WM	-0.127	0.073	.083	0.881 (0.763–1.016)
	IVW	-0.010	0.053	.854	0.991 (0.892–1.099)
RA on DLBCL	MR-Egger	-0.121	0.090	.182	0.885 (0.743–1.055)
	WM	-0.017	0.075	.825	0.983 (0.849–1.138)
	IVW	0.255	0.094	**.007**	1.290 (1.072–1.551)
SS on DLBCL	MR-Egger	0.575	0.151	**.032**	1.776 (1.320–2.390)
	WM	0.357	0.084	**<.001**	1.428 (1.211–1.684)
	IVW	0.143	0.046	**.002**	1.153 (1.053–1.262)
SLE on DLBCL	MR-Egger	0.528	0.304	.157	1.696 (0.935–3.076)
	WM	0.114	0.058	**.049**	1.120 (1.000–1.256)
	IVW	-0.106	0.022	**<.001**	0.899 (0.862–0.938)
T1DM on DLBCL	MR-Egger	-0.075	0.036	**.038**	0.927 (0.865–0.995)
	WM	-0.119	0.035	**.001**	0.887 (0.828–0.950)
	IVW	0.072	0.058	.212	1.074 (0.959–1.203)
UC on DLBCL	MR-Egger	0.500	0.157	**.003**	1.648 (1.210–2.243)
	WM	0.093	0.068	.169	1.097 (0.961–1.252)

Bold indicates *P* < .05.

AS = ankylosing spondylitis, Beta = beta coefficient, CI = confidence interval, DLBCL = diffuse large B-cell lymphoma, IVW = inverse variance weighted, MCTD = mixed connective tissue disease, MR-Egger = Mendelian randomization-Egger regression, MS = multiple sclerosis, OR = odds ratio, *P*val = *P*-value, RA = rheumatoid arthritis, SE = standard error, SLE = systemic lupus erythematosus, SS = Sjögren syndrome, T1DM = type 1 diabetes mellitus, UC = ulcerative colitis, WM = weighted median.

**Table 2:** MR-presso, heterogeneity and pleiotropy test for DLBCL on different ADs by two-sample MR.

Exposure	Heterogeneity test		Pleiotropy test		MR-PRESSO			
	MR-Egger	IVW	SE	*P*val	Raw	Outlier-corrected	Global Test	Distortion Test
AS	0.043	0.052	0.019	.770	0.595	0.003	0.027	0.422
Crohn disease	0.148	0.157	0.053	.426	0.285	NA	NA	0.238
MCTD	0.012	0.002	0.156	.354	0.249	0.046	0.039	0.961
MS	<0.001	<0.001	0.057	.308	0.630	0.467	<0.001	0.211
Pemphigus	0.095	0.123	0.130	.559	0.998	NA	NA	0.204
Psoriasis	<0.001	<0.001	0.024	.137	0.174	0.680	<0.001	0.080
RA	0.002	<0.001	0.019	.131	0.854	0.703	<0.001	0.167
Sicca syndrome	0.533	0.101	0.059	.101	0.053	NA	NA	0.186
SLE	0.617	0.506	0.146	.268	0.021	NA	NA	0.568
T1DM	0.252	0.246	0.011	.273	<0.001	NA	NA	0.229
UC	0.006	<0.001	0.027	.073	0.217	0.689	<0.001	0.130

NA: no outlier were identified, therefore the results for the outlier-corrected MR are set to NA.

AS = ankylosing spondylitis, DLBCL = diffuse large B-cell lymphoma, MCTD = mixed connective tissue disease, MR-PRESSO = Mendelian randomization pleiotropy RESidual sum and outlier, MS = multiple sclerosis, RA = rheumatoid arthritis, SLE = systemic lupus erythematosus, T1DM = type 1 diabetes mellitus, UC = ulcerative colitis.

### 3.3. Sensitivity analyses

Initially, we performed the MR-Egger intercept test on all ADs, with the outcomes displayed in Table 2. The *P*-value for all ADs exceeded .05, suggesting the absence of gene-directed pleiotropy. In addition, the MR-PRESSO global test results showed that no abnormal SNPs (outliers) were found in Crohn disease (NA), Pemphigus (NA), Sicca syndrome (NA), SLE (NA), and T1DM (NA), while the MR-PRESSO global test results of AS (*P*_Global Test_ = .027), mixed connective tissue disease (MCTD) (*P*_Global Test_ = .039), MS (*P*_Global Test_ < .001), psoriasis (*P*_Global Test_ < .001), RA (*P*_Global Test_ < .001), and UC (*P*_Global Test_ < .001) suggested the possibility of pleiotropy. After excluding outliers, the corrected results showed that the corrected results of MS (*P*_Raw_ = .630, *P*_corrected_ = .467), psoriasis (*P*_Raw_ = .174, *P*_corrected_ = .680), RA (*P*_Raw_ = .854, *P*_corrected_ = .703), and UC (*P*_Raw_ = .217, *P*_corrected_ = .689) were the same as the original results and still had no statistical significance, and the MR-PRESSO distortion test results also indicate that there is no difference in the results before and after correction.

**Figure 3.**
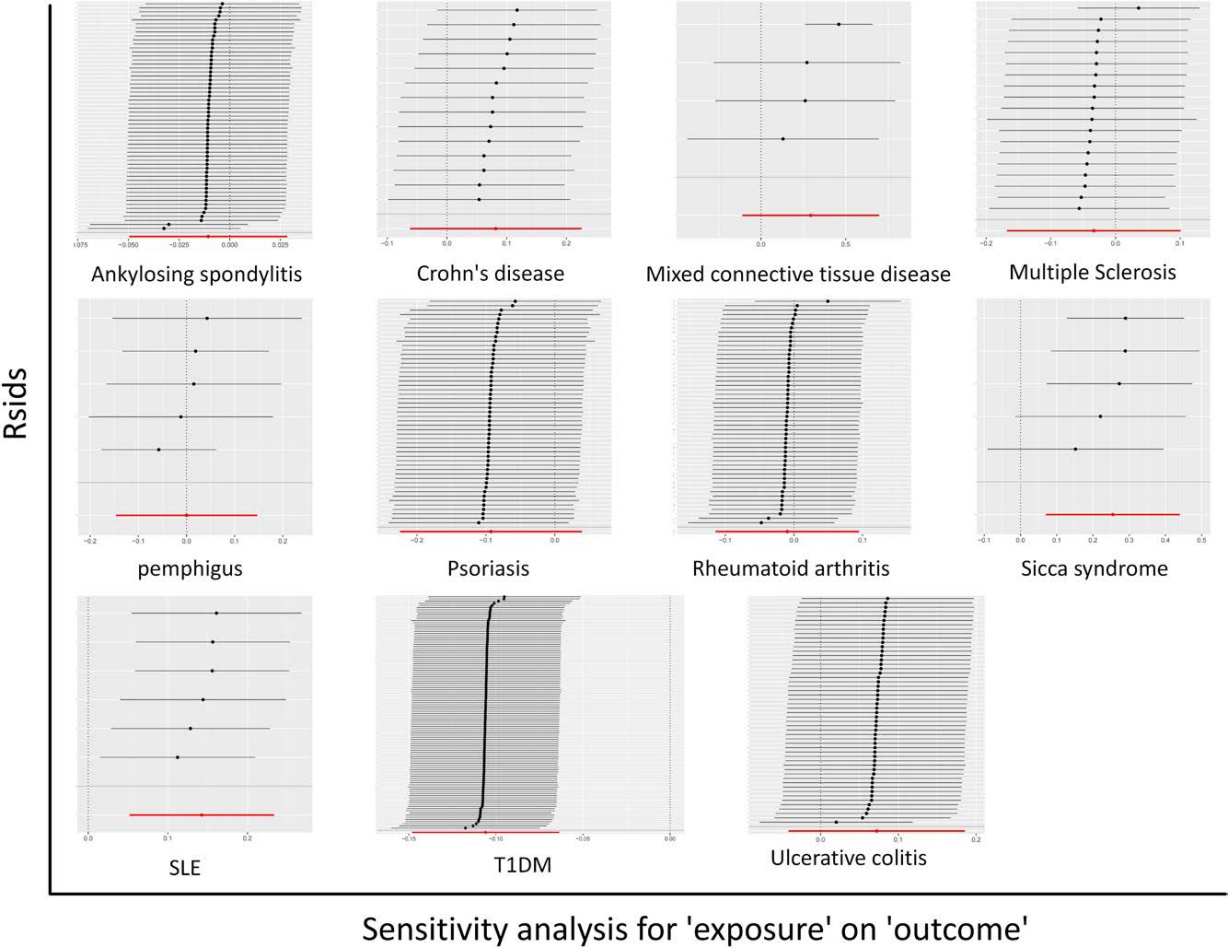
Outcomes of the leave-one-out sensitivity analysis concerning ADs and diffuse large B-cell lymphoma (DLBCL). ADs = autoimmune diseases.

However, as for AS and MCTD, although the *P*-values of AS (*P*_Raw_ = .595, *P*_corrected_ = .003) and MCTD (*P*_Raw_ = .249, *P*_corrected_ = .046) after correcting outliers were both <.05 compared to the original results, the MR-PRESSO distortion test results were still not statistically significant, indicating that there was no difference in the results before and after correction. The Cochran Q heterogeneity test results as shown in Table 2 suggested that no significant heterogeneity was observed in the SS (*P*_MR-Egger_ = .533, *P*_IVW_ = .101), SLE (*P*_MR-Egger_ = .617, *P*_IVW_ = .506), T1DM (*P*_MR-Egger_ = .246, *P*_IVW_ = .246) study. The leave-one-out sensitivity analysis depicted in Figure 3 demonstrated that the causal link between SS, SLE, T1DM, and DLBCL remained consistent and dependable, even with the exclusion of any chosen SNPs. However, the MCTD (*P*_MR-Egger_ = .012, *P*_IVW_ = .002), psoriasis (*P*_MR-Egger_ < .001, *P*_IVW_ < .001), and UC (*P*_MR-Egger_ = .006, *P*_IVW_ < .001) was observed significant heterogeneity in the Cochran Q heterogeneity test. For other ADs like psoriasis, RA, MS, and UC, we primarily utilize fixed-effects models for analysis and statistics due to the variability in Cochran Q test results, ensuring the reliability of the findings, with specific results displayed in Table 2.

## 4. Discussion

### 4.1. Main interpretation

ADs, such as MS, T1DM, and SLE, are caused by a chronic immune response that targets the host’s own cells, tissues, and organs, subsequently leading to tissue destruction, dysfunction, and pathology.^[19]^ Numerous studies indicate a significant link between cancer and autoimmune disorders, yet the exact mechanisms and pathophysiology are not well understood, posing difficulties for disease prevention and treatment.^[20,21]^ Patients suffering from IBD, like UC or Crohn disease, are known to have a higher likelihood of developing colorectal cancer.^[22]^ A systematic review conducted recently pointed out that DLBCL is more common in patients suffering from IBD and is the predominant type of tumor in this demographic.^[23]^ The causal connection between IBD and lymphomas, particularly DLBCL among NHL, has not been thoroughly investigated. In addition, the MR-Egger intercept test results demonstrated no indication of horizontal pleiotropy between UC and Crohn disease, meaning our analysis was not impacted by confounding factors. This suggests that UC is an independent risk factor for DLBCL, regardless of therapeutic strategies.

Recent research indicates that RA patients using the anti-cancer drug methotrexate face a higher risk of developing secondary cancers, especially lymphoma, which is a broadly accepted conclusion.^[20,21,24]^ There is ongoing debate about whether primary RA heightens the risk of developing cancer. A study in South Korea that looked back at a group of 2104 RA patients over an average period of 7.4 years discovered a heightened risk of NHL in those with RA.^[25]^ In Denmark, a study with 3499 RA patients showed no connection between recent-onset or long-term RA and the occurrence of lymphoproliferative cancers after adjusting for confounders. Nevertheless, this cohort was only monitored for a period of 4 years.^[26]^ The data we obtained show no evidence of a causal connection between AS and DLBCL. The present research indicates that the average risk of lymphoma in individuals with AS is similar to that of the general population. Moreover, current research has associated SS and SLE with a heightened risk of several cancers, such as vulvar, lung, thyroid, and potentially liver cancer. Furthermore, there is a common association between SLE and lymphomas, notably the NHL subtype and DLBCL.^[27]^ DLBCL begins in activated lymphocytes, indicating that persistent inflammation might increase the risk of lymphoma in autoimmune conditions like NHL. In line with this, our research found a strong causal link between SS, SLE, and DLBCL, with the findings staying consistent even after using different analytical approaches. Conversely, only case reports have suggested a possible link between pemphigus and Hodgkin lymphoma,^[28]^ but whether pemphigus is related to DLBCL has not been explored, and current studies only suggest that as a subtype of pemphigus, it may be closely related to lymphoma.^[29]^ According to a recent matched cohort study, MS is not linked to an increased risk of lymphoma or higher mortality in those with lymphoma. Our results, consistent with these findings, also demonstrate no causal connection between MS and DLBCL.^[30]^

Our research on psoriasis revealed no significant causal link between psoriasis and DLBCL after removing outliers. Currently, the conclusion regarding whether psoriasis increases the risk of lymphoma remains inconsistent.^[31,32]^ Our findings indicate that the strong link between psoriasis and DLBCL found in earlier research might be affected by confounding variables, like the use of immunosuppressive drugs. Regarding T1DM, a cohort study from Sweden with over 10 years of follow-up found a significant increase in the incidence of gastric cancer, skin cancer, and leukemia among T1DM patients, with the risk not decreasing even after prolonged follow-up.^[33]^ A cohort study conducted on a large population in Japan revealed that patients with T1DM have a notably higher risk of developing gastric, liver, and pancreatic cancers.^[34]^ However, the risk of lymphoma has not been reported yet.

In our MR analysis, we identified a novel inverse association between T1DM and the risk of DLBCL. According to the existing literature, there are currently no epidemiological or clinical studies that have directly reported a clear protective effect of T1DM on DLBCL or other NHL subtypes. The available epidemiological evidence regarding the association between T1DM and NHL, including DLBCL, remains inconclusive.^[35]^ Multiple retrospective studies have emphasized the limited number of investigations on T1DM and specific cancer risks, generally characterized by small sample sizes and substantial heterogeneity in results. Specifically for NHL, the literature consistently categorizes the findings as “inconclusive.”^[35]^ At present, there is no direct mechanistic evidence in the literature to support this potential inverse association. Theoretical explanations remain speculative and primarily involve factors unique to T1DM, such as alterations in the immune microenvironment, chronic hyperglycemia-induced metabolic changes, insulin deficiency, or genetic background, but these largely remain hypothetical and lack direct empirical support. Notably, the risk profile for NHL in individuals with T1DM may differ from that in type 2 diabetes, underscoring the importance of distinguishing between different types of diabetes in relevant research.

Furthermore, none of the studies included in the review reported a statistically significant reduction in NHL incidence or mortality among T1DM populations.^[35]^ Most studies did not observe a relevant association or had insufficient data to draw robust conclusions.^[35]^ Therefore, there is an urgent need for large-scale epidemiological studies with clear diabetes type stratification and mechanistic experimental investigations to validate our findings and further elucidate the underlying biological basis. When interpreting results, it is also necessary to consider the potential influences of exogenous insulin therapy and genetic background. In summary, our study offers a novel perspective on the relationship between T1DM and lymphoma; however, caution is warranted before generalizing the conclusions, and further studies are needed to obtain more definitive scientific evidence.

As far as we know, this is the initial two-sample MR study offering fresh perspectives on the causal link between various ADs and DLBCL. Using MR methods aids in minimizing bias from confounding variables and provides a strong estimation of causal effects. Additionally, we employed basic methods to explore potential pleiotropy in the IVs, and by correcting for pleiotropy, we enhanced the reliability of the MR analysis.

### 4.2. Limitations

However, this study has some limitations. Observational studies have initially suggested that particular treatments for ADs could heighten the risk of developing malignant lymphoma. The absence of GWAS data on ADs treatments prevented the verification of causal relationships through MR in this study, emphasizing the necessity for future GWAS and MR research. Second, a limitation of our study is the inability to stratify the analysis by factors such as immune classification, disease severity, gender, and age of DLBCL. Thirdly, because the study primarily involves a European population, the causal relationship between different ADs and DLBCL in other demographics is not yet clear. Ultimately, even though we employed different techniques to eliminate outliers, we cannot entirely dismiss the influence of horizontal pleiotropy on our findings.

### 4.3. Conclusion

In summary, our MR investigation presents evidence that certain autoimmune conditions, like SS, SLE, UC, and T1DM, are significantly associated with DLBCL. Nonetheless, our findings do not indicate a causal link between DLBCL and AS, Crohn disease, pemphigus, psoriasis, or RA. Some of the associations between ADs and DLBCL observed in previous studies may be influenced by confounding factors.

## Author contributions

**Project administration:** Tian Li.

**Supervision:** Yu Li.

**Validation:** Tianhao Wang.

**Writing – original draft:** Jiaying Duan.

**Writing – review & editing:** Litian Ma.

## Supplementary Material


