# Supplementary material for: Autoimmune diseases and diffuse large B-cell lymphoma: A Mendelian randomization study
Source: Medicine (Baltimore). 2025 Jun 20;104(25):e42855. doi: 10.1097/MD.0000000000042855 (PMC12187306; doi:10.1097/MD.0000000000042855)
Supplement: Supplementary file 1 [file medi-104-e42855-s001.pdf]

Supplementary Table 1. Details of data source included in the study.

Phenotypes	year	Data source	Number of SNPs	Cases	Controls
DLBCL	2022	FinnGen	20,167,520	1010	287137
Ankylosing spondylitis	2022	FinnGen	20,372,921	2860	270964
Crohn's disease	2022	FinnGen	20,169,882	2007	359927
Mixed connective tissue disease	2022	FinnGen	20170236	1849	375428
Multiple Sclerosis	2019	International Multiple Sclerosis Genetics Consortium	6,304,359	47,429	68,374
pemphigus	2022	FinnGen	20170203	162	375767
Psoriasis	2018	MRC-IEU	9,851,867	5,314	457,619
RA	2022	FinnGen	20165764	12555	240862
Sicca syndrome	2022	FinnGen	16,380,454	2495	365533
SLE	2021	ebi	24,198,877	1023	281127
T1DM	2021	FinnGen	20,168,683	8967	308373
Ulcerative colitis	2021	FinnGen	16,380,466	5034	371530

Supplementary Table 2 The baseline characteristics of the selected SNPs in the AS GWAS.

SNP	OA	EA	Beta	Se	EAF	P-value
rs72749142	G	A	-0.235299	0.0411114	0.138556	1.04E-08
rs6759003	T	C	-0.187972	0.0276852	0.662385	1.12E-11
rs2032890	A	C	-0.243199	0.0312391	0.268649	6.97E-15
rs149679145	A	G	0.46119	0.0681402	0.0296196	1.30E-11
rs66854396	A	G	-0.173126	0.0301978	0.281844	9.86E-09
rs79151153	G	T	0.423919	0.0472634	0.0660826	2.98E-19
rs10946660	A	C	0.61315	0.0493202	0.0537117	1.75E-35
rs72833065	T	C	0.717259	0.0615127	0.0313235	2.03E-31
rs2817213	G	A	-0.160478	0.0278504	0.366492	8.31E-09
rs77831243	T	C	0.847808	0.0683531	0.0232228	2.51E-35
rs9467434	G	T	0.169698	0.0302079	0.703315	1.94E-08
rs9358842	C	G	0.369628	0.065317	0.033945	1.52E-08
rs2254974	T	C	-0.273826	0.0353802	0.856887	9.98E-15
rs12195837	C	T	0.329852	0.0398483	0.104153	1.26E-16
rs62394296	C	T	0.370563	0.034988	0.138379	3.28E-26
rs3884392	G	T	0.467401	0.0312653	0.173856	1.57E-50
rs2223591	G	A	0.740011	0.0520715	0.0425817	7.78E-46
rs35183513	G	A	0.524058	0.0335985	0.135403	7.55E-55
rs2142686	T	C	-0.203538	0.0289636	0.324597	2.10E-12
rs190364070	C	T	1.40081	0.0373011	0.0531805	0
rs1794587	A	C	0.37826	0.0260899	0.432069	1.24E-47
rs13195617	A	T	0.62471	0.0263192	0.267754	1.54E-124
rs9261744	C	T	-0.426322	0.032845	0.242231	1.59E-38
rs201277281	G	A	0.834553	0.146791	0.00574	1.31E-08
rs2286656	C	T	0.762057	0.0264428	0.222621	1.24E-182
rs55640410	C	G	-0.44453	0.0640044	0.058718	3.78E-12
rs3734869	G	A	1.0404	0.024415	0.217409	0
rs3873387	C	A	0.918977	0.0241108	0.27032	0
rs9265884	T	C	0.960022	0.0239326	0.271986	0
rs2859448	C	T	-0.82959	0.0256598	0.765845	2.62E-229
rs510564	T	C	0.637274	0.0342176	0.117657	2.05E-77
rs2854260	C	T	0.305795	0.0390049	0.109635	4.51E-15
rs144614916	A	C	1.01621	0.0856804	0.0127767	1.90E-32
rs9275709	G	A	-0.3919	0.0358342	0.194742	7.71E-28
rs144112342	T	C	1.22962	0.0657006	0.018476	3.70E-78
rs206763	G	A	1.24968	0.0715375	0.015478	2.47E-68
rs1126513	G	T	0.380871	0.0326393	0.163489	1.83E-31
rs3117034	T	A	0.331725	0.0262161	0.406353	1.07E-36
rs4713650	C	T	-0.371735	0.0260022	0.580012	2.31E-46
rs12194518	C	G	0.513108	0.0385557	0.101237	2.07E-40
rs9368778	A	T	-0.304062	0.0401545	0.145663	3.67E-14

rs2499762	C	T	0.250837	0.0266377	0.378637	4.66E-21
rs2495996	C	T	0.169629	0.0266037	0.46451	1.82E-10
rs139808058	G	C	0.95314	0.0634097	0.0249137	4.57E-51
rs10807156	T	A	0.235876	0.0284512	0.281198	1.13E-16
rs7751919	G	A	0.316986	0.0471604	0.0718951	1.80E-11
rs138270309	G	A	0.288961	0.0441491	0.0866549	5.94E-11
rs186418170	A	T	0.506726	0.0868388	0.0195308	5.37E-09
rs7805568	G	A	-0.338929	0.0489351	0.934141	4.33E-12
rs35887628	G	C	0.31032	0.0458323	0.0778009	1.28E-11
rs55859346	G	C	0.291993	0.0347178	0.152768	4.08E-17
rs10224849	G	C	0.23062	0.0405547	0.107929	1.30E-08
rs181316459	G	C	0.6658	0.0522059	0.0471767	2.99E-37
rs62443225	G	A	0.431922	0.0444618	0.0778205	2.62E-22
rs35978646	A	T	0.242829	0.0350238	0.15603	4.11E-12
rs142695953	C	A	0.214716	0.0329683	0.186506	7.38E-11
rs76924796	G	A	0.324191	0.059284	0.0443542	4.54E-08

AS: Ankylosing spondylitis; SNP: single nucleotide polymorphism; beta: beta coefficient for effect allele; Se: standard error for effect allele; EA: effect allele; OA: other allele; EA: effect allele frequency.

Supplementary Table 3. The baseline characteristics of the selected SNPs in the Crohn's disease GWAS.

SNP	OA	EA	Beta	Se	EAF	P-value
rs2902440	G	A	-0.214049	0.0318299	0.444661	1.76E-11
rs4631223	A	G	0.202164	0.0321118	0.568743	3.06E-10
rs2734975	C	A	0.185841	0.0313602	0.475445	3.10E-09
rs9270559	T	G	0.181164	0.0315331	0.432791	9.18E-09
rs116929608	A	G	0.324005	0.0551678	0.0734177	4.28E-09
rs34616999	C	G	0.182484	0.032046	0.378503	1.24E-08
rs10807943	T	C	-0.372907	0.0583869	0.936786	1.69E-10
rs6966019	T	C	-0.230456	0.0334555	0.69829	5.64E-12
rs181316459	G	C	0.598315	0.0634037	0.0467176	3.85E-21
rs62443225	G	A	0.415374	0.0531221	0.0773303	5.31E-15
rs35978646	A	T	0.228495	0.0416744	0.156097	4.18E-08
rs4979462	C	T	0.330675	0.0514955	0.0860817	1.35E-10
rs7079205	C	T	0.176571	0.0321707	0.367618	4.05E-08
rs1332099	T	C	-0.171554	0.0314383	0.540745	4.85E-08

SNP: single nucleotide polymorphism; beta: beta coefficient for effect allele; Se: standard error for effect allele; EA: effect allele; OA: other allele; EA: effect allele frequency.

Supplementary Table 4. The baseline characteristics of the selected SNPs in the Mixed connective tissue disease GWAS.

SNP	OA	EA	Beta	Se	EAF	P-value
rs2923008	G	T	0.521316	0.049098	0.0910875	2.46E-26
rs727939	A	G	0.21074	0.032863	0.414607	1.43E-10
rs113708239	C	T	0.289652	0.039716	0.184029	3.03E-13
rs17153419	A	G	0.215352	0.034186	0.325058	2.99E-10

SNP: single nucleotide polymorphism; beta: beta coefficient for effect allele; Se: standard error for effect allele; EA: effect allele; OA: other allele; EA: effect allele frequency.

Supplementary Table 5. The baseline characteristics of the selected SNPs in the Multiple Sclerosis GWAS.

SNP	OA	EA	P-value	Beta	Se	EAF
rs142869490	G	A	3.46E-12	0.718851	0.103321	0.013308
rs137878408	G	C	1.48E-13	0.772016	0.10448	0.012574
rs76761992	C	A	7.79E-09	0.321859	0.0557532	0.065967
rs9380141	T	C	2.05E-24	-0.326159	0.0319871	0.367565
rs147023494	C	T	1.38E-14	0.505288	0.0656379	0.040195
rs183697542	C	T	5.56E-27	0.932637	0.0867091	0.016883
rs2905758	T	C	2.43E-13	0.34886	0.0476405	0.090219
rs35989721	T	C	4.85E-12	-0.376532	0.0544925	0.104295
rs75518104	C	T	4.39E-28	0.726922	0.0661586	0.033897
rs56111157	G	A	7.29E-16	0.42547	0.0527517	0.076032
rs115797804	G	T	9.91E-19	-0.45788	0.0518194	0.124253
rs145365399	A	G	1.03E-11	-0.801344	0.11781	0.029023
rs9271069	A	G	8.00E-130	-0.830626	0.0342637	0.86079
rs36229731	A	C	3.20E-22	-0.390502	0.0402838	0.205816
rs55869430	A	G	1.05E-09	-0.365967	0.0599767	0.087641
rs2071349	C	G	3.98E-11	-0.30178	0.0456914	0.145956
rs62398913	C	T	2.47E-11	0.425757	0.0637835	0.043904
rs8045630	T	C	4.33E-08	-0.167182	0.0305253	0.579457
rs10421112	C	T	3.75E-08	0.171548	0.0311782	0.359908
rs11666265	C	T	2.44E-09	-0.213186	0.0357383	0.25777

SNP: single nucleotide polymorphism; beta: beta coefficient for effect allele; Se: standard error for effect allele; EA: effect allele; OA: other allele; EA: effect allele frequency.

Supplementary Table 6. The baseline characteristics of the selected SNPs in the pemphigus GWAS.

SNP	OA	EA	Beta	Se	EAF	P-value
rs4656837	C	T	-0.49807	0.101188	0.604445	8.56E-07
rs76443979	G	A	0.785192	0.163863	0.066037	1.65E-06
rs184747938	G	A	2.60109	0.554366	0.001464	2.71E-06
rs118174171	T	C	1.10052	0.229267	0.024847	1.59E-06
rs369761389	A	G	1.57846	0.297564	0.010669	1.13E-07

SNP: single nucleotide polymorphism; beta: beta coefficient for effect allele; Se: standard error for effect allele; EA: effect allele; OA: other allele; EA: effect allele frequency.

Supplementary Table 7. The baseline characteristics of the selected SNPs in the psoriasis GWAS.

SNP	OA	EA	Beta	Se	EAF	P-value
rs7542079	T	C	0.0950954	0.0150806	0.561867	2.87E-10
rs80174646	G	T	-0.227133	0.0382237	0.045716	2.81E-09
rs10193310	G	A	0.126764	0.0170155	0.248028	9.34E-14
rs11679753	A	G	-0.091266	0.0155432	0.64863	4.31E-09
rs78456138	C	T	-0.364637	0.0590482	0.0203786	6.61E-10
rs847	T	C	0.126621	0.0157392	0.63499	8.63E-16
rs74817271	G	A	0.224803	0.0267321	0.0744382	4.12E-17
rs6886974	A	T	0.315361	0.0414177	0.0278619	2.65E-14
rs6556423	C	T	-0.180408	0.0152621	0.641772	3.05E-32
rs13153019	T	C	0.101863	0.0164997	0.272931	6.67E-10
rs197997	C	T	-0.142115	0.0238932	0.8996	2.72E-09
rs115086705	C	T	0.248054	0.0379018	0.0345275	5.96E-11
rs9358905	T	A	-0.089533	0.0148744	0.509321	1.75E-09
rs142948774	A	G	0.415131	0.0362696	0.0340322	2.47E-30
rs9461362	C	T	0.171305	0.0200381	0.15009	1.24E-17
rs73400551	T	C	0.201026	0.0291957	0.0629051	5.76E-12
rs116490552	C	T	0.392457	0.0619929	0.0116932	2.44E-10
rs9258033	T	C	0.332195	0.0589717	0.0132752	1.77E-08
rs1655901	T	C	0.267928	0.0147773	0.469909	1.81E-73
rs9391803	T	A	-0.166453	0.0181208	0.233752	4.09E-20
rs74787141	T	C	0.378668	0.0247416	0.0788299	7.09E-53
rs13210419	G	A	0.802509	0.0248769	0.0589556	2.61E-228
rs28367848	C	T	0.474095	0.0159931	0.232926	4.09E-193
rs115457991	G	A	0.401407	0.0318546	0.0461438	2.08E-36
rs73728584	C	A	-0.174234	0.017894	0.243076	2.10E-22
rs149815433	C	T	-0.246381	0.0402043	0.0410653	8.89E-10
rs2736188	G	A	-0.183752	0.0225396	0.137776	3.57E-16
rs9273060	A	T	0.218969	0.0165972	0.249332	9.61E-40
rs9274852	G	A	0.187594	0.0163173	0.278422	1.37E-30
rs3213483	G	T	0.13348	0.0150365	0.56097	6.86E-19
rs12190787	G	C	0.100238	0.0167647	0.256507	2.24E-09
rs9277339	G	A	0.231906	0.0217701	0.119144	1.70E-26
rs4713637	G	C	0.0981278	0.0157317	0.330827	4.44E-10
rs33980500	C	T	0.237708	0.0268401	0.0724488	8.26E-19
rs674451	T	C	0.128161	0.015488	0.344313	1.29E-16
rs9346778	C	T	-0.116749	0.0206424	0.163996	1.55E-08
rs6956343	C	T	0.122643	0.020285	0.152993	1.48E-09
rs181316459	G	C	0.330442	0.0326143	0.0473913	3.99E-24
rs62443225	G	A	0.199119	0.0268994	0.0780019	1.34E-13
rs35978646	A	T	0.113259	0.0202697	0.156686	2.30E-08
rs60600003	T	G	0.149572	0.0236681	0.102104	2.62E-10

rs16903065	C	A	-0.141275	0.0241631	0.115532	5.01E-09
rs2664280	A	T	0.086808	0.0149498	0.451869	6.37E-09
rs1250566	G	A	-0.088896	0.0153274	0.407776	6.64E-09
rs76741620	A	G	0.174314	0.030468	0.0579356	1.06E-08
rs653169	A	G	-0.086189	0.0151004	0.570235	1.14E-08
rs7310615	C	G	-0.086575	0.0150881	0.586418	9.58E-09
rs8904	G	A	-0.126143	0.0154202	0.388059	2.83E-16
rs2021511	C	T	-0.108961	0.0172224	0.265161	2.50E-10
rs144651842	G	A	0.171788	0.0268541	0.0776597	1.58E-10
rs28998802	G	A	0.160654	0.0186763	0.184254	7.83E-18
rs34536443	G	C	-0.281948	0.0474089	0.030271	2.73E-09
rs2769979	T	C	-0.095714	0.0151474	0.604978	2.64E-10

SNP: single nucleotide polymorphism; beta: beta coefficient for effect allele; Se: standard error for effect allele; EA: effect allele; OA: other allele; EA: effect allele frequency.

Supplementary Table 8. The baseline characteristics of the selected SNPs in the rheumatoid arthritis GWAS.

SNP	OA	EA	Beta	Se	EAF	P-value
rs78248443	C	T	-0.178636	0.0262275	0.0752716	9.69E-12
rs12040903	G	A	-0.088286	0.015786	0.2352	2.24E-08
rs1217238	G	A	-0.084332	0.0147355	0.28697	1.05E-08
rs2476601	A	G	-0.348863	0.0171448	0.851574	4.83E-92
rs56175143	G	A	0.275258	0.028435	0.0493133	3.66E-22
rs4274624	C	T	-0.111284	0.0153928	0.76852	4.84E-13
rs3087243	G	A	-0.10625	0.0141899	0.330008	7.01E-14
rs7731626	G	A	-0.108014	0.0149385	0.278813	4.81E-13
rs952579	G	A	0.0919843	0.0154963	0.225654	2.92E-09
rs77831243	T	C	0.240919	0.0419555	0.0231086	9.34E-09
rs9461153	G	A	-0.098589	0.0168201	0.196314	4.59E-09
rs76064269	G	A	0.129604	0.020833	0.107111	4.94E-10
rs144952788	A	G	0.441159	0.0308501	0.0381064	2.19E-46
rs9295747	T	G	0.129241	0.0137509	0.337684	5.52E-21
rs116466121	A	T	0.16594	0.0173167	0.16178	9.46E-22
rs9391630	A	G	0.190142	0.0141617	0.290064	4.23E-41
rs9501030	T	A	0.137686	0.0159828	0.20214	7.02E-18
rs115749638	C	T	-0.221059	0.035743	0.0388641	6.22E-10
rs1265109	G	T	0.140117	0.0139551	0.315682	1.01E-23
rs9265521	G	A	0.153262	0.0143687	0.681349	1.46E-26
rs114291795	C	G	0.563252	0.0215378	0.0761127	9.40E-151
rs3093998	C	A	0.254986	0.0149501	0.710344	3.16E-65
rs62395789	G	A	-0.193281	0.0326258	0.0483228	3.14E-09
rs114484678	T	C	0.303086	0.0245518	0.0648858	5.20E-35
rs41291794	A	T	-0.235087	0.0332364	0.046186	1.51E-12
rs71538573	T	A	0.474949	0.0633293	0.0088794	6.40E-14
rs114335056	A	G	0.623384	0.016035	0.153695	0
rs28579922	G	A	-0.539175	0.0223116	0.124716	5.11E-129
rs17427887	G	T	0.327824	0.0450688	0.018239	3.49E-13
rs1383258	C	T	0.503364	0.0254443	0.0556039	4.17E-87
rs2071541	A	G	-0.263672	0.0180547	0.177172	2.65E-48
rs144112342	T	C	0.805776	0.0389825	0.0186196	6.43E-95
rs620202	G	T	0.195814	0.0131293	0.443214	2.66E-50
rs206763	G	A	0.788829	0.0428364	0.0155008	9.97E-76
rs13203715	A	G	-0.230409	0.0214843	0.116399	7.81E-27
rs6934244	C	A	0.311298	0.0268045	0.0553512	3.51E-31
rs2856822	A	C	-0.26097	0.0156133	0.254238	1.03E-62
rs2395450	C	T	0.186495	0.0134652	0.57271	1.27E-43
rs55806967	G	T	-0.166912	0.0261727	0.0758384	1.80E-10
rs62399315	G	A	0.26546	0.0389224	0.0255218	9.09E-12
rs2495969	G	T	0.136814	0.0211623	0.882679	1.01E-10

rs6456160	T	C	-0.079268	0.0132719	0.556647	2.34E-09
rs74611185	C	A	0.107229	0.0196439	0.124275	4.80E-08
rs3757387	T	C	0.0913903	0.0132944	0.424981	6.23E-12
rs16903065	C	A	-0.143169	0.0213703	0.11551	2.09E-11
rs3118470	T	C	0.0843758	0.0132524	0.441546	1.93E-10
rs10821948	C	A	0.0760685	0.0133917	0.425718	1.34E-08
rs548877	A	G	0.0964878	0.0170291	0.176204	1.46E-08
rs11513729	C	T	0.0978485	0.0135079	0.381047	4.36E-13
rs8002731	A	C	-0.090278	0.0138528	0.362902	7.18E-11
rs34536443	G	C	-0.308343	0.0423988	0.030152	3.53E-13
rs6065926	A	G	0.08477	0.0149909	0.728706	1.56E-08

SNP: single nucleotide polymorphism; beta: beta coefficient for effect allele; Se: standard error for effect allele; EA: effect allele; OA: other allele; EA: effect allele frequency.

Supplementary Table 9. The baseline characteristics of the selected SNPs in the sjogren's syndrome GWAS.

SNP	OA	EA	Beta	Se	EAF	P-value
rs10174238	A	G	-0.204598	0.0323563	0.764432	2.56E-10
rs150724213	A	G	0.450504	0.0751008	0.0286522	1.99E-09
rs2004640	G	T	-0.24807	0.0283561	0.476859	2.16E-18
rs3117581	G	A	0.733315	0.0397579	0.0912756	5.77E-76
rs9272305	G	C	0.420946	0.0335896	0.178178	4.99E-36

SNP: single nucleotide polymorphism; beta: beta coefficient for effect allele; Se: standard error for effect allele; EA: effect allele; OA: other allele; EA: effect allele frequency.

Supplementary Table 10. The baseline characteristics of the selected SNPs in the systemic lupus erythematosus GWAS.

SNP	OA	EA	Beta	Se	EAF	P-value
rs2734961	C	T	0.568708	0.0811236	0.0911048	2.38E-12
rs3095339	A	G	0.536233	0.0623476	0.181564	7.92E-18
rs2253040	G	T	0.363972	0.0557793	0.313519	6.79E-11
rs9268671	A	G	-0.529024	0.0525346	0.634791	7.50E-24
rs9273324	C	T	0.50462	0.0615215	0.197623	2.36E-16
rs1042151	A	G	0.414173	0.0623081	0.199669	2.99E-11

SNP: single nucleotide polymorphism; beta: beta coefficient for effect allele; Se: standard error for effect allele; EA: effect allele; OA: other allele; EA: effect allele frequency.

Supplementary Table 11. The baseline characteristics of the selected SNPs in the type 1 diabetes mellitus GWAS.

SNP	OA	EA	Beta	Se	EAF	P-value
rs111937080	T	C	0.241496	0.0427436	0.0297524	1.61E-08
rs11102634	C	A	0.229849	0.0394117	0.0377392	5.48E-09
rs1217238	G	A	-0.098452	0.0174072	0.287742	1.55E-08
rs2476601	A	G	-0.44637	0.0196745	0.851736	5.92E-114
rs56175143	G	A	0.363564	0.0329596	0.0487628	2.72E-28
rs61819249	G	A	0.231376	0.0391999	0.0353176	3.58E-09
rs115204205	T	C	0.264625	0.0419177	0.0311899	2.74E-10
rs1990760	C	T	0.106056	0.0158947	0.585658	2.52E-11
rs138242238	C	T	-0.154667	0.0275004	0.0962184	1.86E-08
rs3087243	G	A	-0.168952	0.0167364	0.329841	5.82E-24
rs35914000	G	A	0.0871909	0.0158683	0.39213	3.91E-08
rs9391997	A	G	0.103025	0.0152991	0.474402	1.65E-11
rs199026	A	G	-0.120223	0.0212918	0.851202	1.64E-08
rs115708172	C	A	0.610371	0.0582562	0.0118745	1.10E-25
rs1511478	C	T	0.215294	0.025879	0.0858588	8.85E-17
rs9467015	G	A	0.0953218	0.0157989	0.430247	1.61E-09
rs7775357	G	A	-0.098877	0.0157028	0.406913	3.04E-10
rs1742283	G	C	-0.282803	0.0388183	0.966196	3.21E-13
rs807509	C	G	0.139136	0.0161125	0.333658	5.86E-18
rs9467329	G	C	0.738719	0.134143	0.0021886	3.65E-08
rs9379697	A	T	0.149105	0.016141	0.320437	2.52E-20
rs35398163	G	A	0.245278	0.0349051	0.0436625	2.11E-12
rs2744306	A	C	-0.11131	0.0155835	0.593425	9.14E-13
rs72831263	G	A	-0.12723	0.0175191	0.274568	3.80E-13
rs13217797	T	C	0.261236	0.0180754	0.206662	2.42E-47
rs62393716	C	T	-0.453022	0.0514384	0.0306608	1.28E-18
rs111706643	G	A	-0.413657	0.0452979	0.0383802	6.73E-20
rs142869490	G	A	-0.435353	0.0774596	0.0132801	1.91E-08
rs151076348	C	G	0.191016	0.0342923	0.0473617	2.54E-08
rs62402048	T	G	0.394506	0.0652235	0.0111667	1.46E-09
rs114513157	C	T	-0.21939	0.0224457	0.15388	1.45E-22
rs75165919	G	A	0.477181	0.0251458	0.0777997	2.66E-80
rs78620132	T	C	-0.344033	0.0427099	0.0412298	7.94E-16
rs3129123	T	C	-0.249807	0.0154215	0.614463	5.16E-59
rs17179220	G	A	-0.578194	0.04549	0.0401429	5.18E-37
rs9261408	C	T	-0.445681	0.0304936	0.0862707	2.24E-48
rs111455094	T	C	0.470924	0.0568186	0.0139308	1.15E-16
rs115855851	A	G	-0.497391	0.0709562	0.0161848	2.39E-12
rs78868683	G	C	0.547578	0.0411309	0.0269312	1.94E-40
rs146353770	T	A	-0.475966	0.0831193	0.0119245	1.03E-08
rs115194949	G	A	0.738391	0.0384943	0.0252691	5.25E-82

rs145162876	A	T	-0.654573	0.0868556	0.011083	4.83E-14
rs1265090	G	A	0.558781	0.0195215	0.134267	3.39E-180
rs28397274	A	G	0.403236	0.0159562	0.28015	6.59E-141
rs17192533	C	G	-0.367319	0.0224745	0.160874	4.81E-60
rs2596544	T	A	0.617023	0.0160334	0.226584	0
rs9266801	G	A	0.686175	0.037003	0.0282473	9.16E-77
rs17206980	C	T	-0.468832	0.0405478	0.0496754	6.39E-31
rs17201144	A	G	-0.615736	0.0390772	0.0564195	6.16E-56
rs1053924	T	C	0.621513	0.0162569	0.635504	0
rs116519179	G	A	-0.758055	0.0728742	0.018483	2.42E-25
rs2073044	C	T	0.708466	0.0159279	0.21742	0
rs116353692	A	C	0.664072	0.0378857	0.0285089	8.71E-69
rs35656734	C	T	-0.518932	0.0155769	0.425709	2.40E-243
rs1383258	C	T	1.40311	0.0221231	0.0541405	0
rs41316552	T	C	-0.372628	0.0228511	0.155774	8.83E-60
rs114311872	C	T	-0.585448	0.0408171	0.0497914	1.18E-46
rs75439840	G	C	0.596571	0.0367011	0.0313663	2.06E-59
rs1063478	C	T	-0.203619	0.0267338	0.100711	2.61E-14
rs9277182	A	G	0.383052	0.0239311	0.859271	1.15E-57
rs9277194	C	T	0.442561	0.0150896	0.419376	4.44E-189
rs2294474	G	T	-0.354328	0.0158541	0.400337	1.23E-110
rs144155310	A	C	1.15731	0.0674165	0.0062909	4.73E-66
rs9394158	G	T	0.303441	0.016276	0.282781	1.43E-77
rs11753213	A	G	0.351464	0.0151588	0.400676	6.38E-119
rs142776031	G	A	0.305782	0.0420696	0.0293853	3.64E-13
rs6902545	G	A	0.192185	0.0158032	0.356011	5.01E-34
rs57786715	G	A	-0.345289	0.0220948	0.165618	4.72E-55
rs192061534	G	A	0.451248	0.0676774	0.0100268	2.60E-11
rs114444048	G	A	0.801728	0.0434974	0.0195005	7.32E-76
rs1565364	G	C	-0.175052	0.0169865	0.738575	6.66E-25
rs56097866	C	T	-0.270446	0.0221726	0.159522	3.21E-34
rs3846869	C	A	0.170932	0.0153564	0.456244	8.86E-29
rs3798544	G	A	0.123754	0.0193171	0.185527	1.49E-10
rs13213430	G	A	0.339587	0.0529345	0.0173852	1.41E-10
rs7760951	T	C	-0.114513	0.020752	0.846518	3.43E-08
rs880062	C	T	0.13479	0.0161294	0.326438	6.44E-17
rs71569312	C	T	-0.310552	0.0548201	0.0241747	1.47E-08
rs146627469	G	C	0.4021	0.0685558	0.0099683	4.48E-09
rs34405021	T	G	-0.188222	0.0299015	0.085833	3.08E-10
rs1742139	T	A	0.217994	0.0264083	0.892527	1.52E-16
rs113300080	C	A	0.296104	0.0543041	0.0159948	4.96E-08
rs6908626	G	T	0.180912	0.0229956	0.115306	3.63E-15
rs1591805	A	G	0.0890258	0.0153436	0.451038	6.55E-09
rs181316459	G	C	-0.22258	0.0400223	0.0476821	2.68E-08

rs1872424	T	C	-0.09069	0.0157945	0.429843	9.36E-09
rs7823699	A	G	-0.095007	0.0163794	0.666735	6.62E-09
rs7034200	C	A	0.0901234	0.0155537	0.497391	6.86E-09
rs61832754	A	G	-0.312023	0.0534778	0.0260141	5.39E-09
rs7090530	C	A	0.134216	0.0168467	0.675477	1.63E-15
rs35947132	G	A	-0.268058	0.0457191	0.034184	4.54E-09
rs60888743	A	G	-0.104384	0.0187815	0.230404	2.73E-08
rs7130222	T	G	-0.100921	0.0180637	0.261583	2.31E-08
rs113257255	C	G	0.29267	0.0531144	0.0192582	3.58E-08
rs689	A	T	0.460279	0.0206414	0.790874	3.79E-110
rs1476495	G	C	-0.249262	0.0326775	0.0712955	2.39E-14
rs4755391	G	T	-0.10193	0.0181189	0.256705	1.85E-08
rs1131017	C	G	-0.15485	0.0155976	0.578482	3.15E-23
rs3184504	T	C	-0.151947	0.0156567	0.591357	2.87E-22
rs34337125	G	A	-0.123605	0.0159171	0.417174	8.13E-15
rs2289702	C	T	-0.131116	0.0239844	0.127651	4.58E-08
rs12928537	G	A	-0.11948	0.0172469	0.302242	4.28E-12
rs151233	C	T	0.136321	0.0229046	0.12325	2.65E-09
rs144517729	T	A	0.184161	0.0321043	0.0585812	9.67E-09
rs2847273	A	C	-0.10445	0.0157564	0.438707	3.38E-11
rs425105	T	C	-0.122034	0.0210787	0.171673	7.06E-09
rs281379	G	A	0.105862	0.0157047	0.408956	1.58E-11
rs3788013	C	A	0.117317	0.0162309	0.34395	4.90E-13
rs74203920	C	T	0.314781	0.0383473	0.0369202	2.24E-16
rs713875	C	G	0.126805	0.0155221	0.477681	3.10E-16

SNP: single nucleotide polymorphism; beta: beta coefficient for effect allele; Se: standard error for effect allele; EA: effect allele; OA: other allele; EA: effect allele frequency.

Supplementary Table 12. The baseline characteristics of the selected SNPs in the Ulcerative colitis GWAS.

SNP	OA	EA	EAF	Beta	Se	P-value
rs12736494	A	G	0.264236	-0.137666	0.0232453	3.17E-09
rs4655215	C	T	0.764144	-0.148334	0.0230026	1.13E-10
rs10737481	G	T	0.509662	0.177217	0.0200527	9.78E-19
rs6426749	C	G	0.0897285	-0.210799	0.0372527	1.53E-08
rs11209026	A	G	0.0456921	-0.382291	0.0536539	1.04E-12
rs7551957	C	T	0.446144	-0.15757	0.0203277	9.08E-15
rs6427868	G	A	0.221321	-0.159913	0.0249333	1.42E-10
rs3024495	T	C	0.157263	0.262195	0.025977	5.91E-24
rs4845140	T	C	0.056491	-0.307627	0.0476677	1.09E-10
rs1878668	G	T	0.595569	0.119883	0.0205999	5.90E-09
rs13024106	A	G	0.583289	-0.115902	0.0202057	9.69E-09
rs34236350	T	C	0.267419	0.17698	0.0221354	1.29E-15
rs3197999	A	G	0.391682	0.184657	0.0202772	8.50E-20
rs13165038	C	T	0.312207	-0.133349	0.0221051	1.61E-09
rs115535082	A	G	0.0143327	0.409415	0.0745426	3.97E-08
rs1264711	A	G	0.260903	-0.17573	0.0234588	6.83E-14
rs147168394	A	G	0.0147893	0.487218	0.0712775	8.17E-12
rs3104412	G	A	0.463013	-0.150948	0.02009	5.75E-14
rs4587163	T	C	0.284129	-0.264094	0.0229326	1.09E-30
rs1042131	A	C	0.501915	-0.137989	0.0200402	5.75E-12
rs2296336	G	C	0.366827	-0.122417	0.0210435	5.98E-09
rs117954350	A	G	0.0269328	0.398014	0.0552147	5.66E-13
rs60810442	T	C	0.193761	0.152605	0.024683	6.31E-10
rs146249753	T	C	0.121628	0.184629	0.0296123	4.52E-10
rs7805568	A	G	0.934292	-0.332397	0.0370819	3.14E-19
rs7781433	A	G	0.0744673	0.287839	0.0352755	3.36E-16
rs6956343	T	C	0.152998	0.217582	0.0266856	3.53E-16
rs10224849	C	G	0.108121	0.21354	0.0306418	3.19E-12
rs6974185	A	G	0.685669	-0.143825	0.0213859	1.75E-11
rs181316459	C	G	0.0473934	0.597776	0.0401735	4.45E-50
rs62443225	A	G	0.0779957	0.40874	0.0338188	1.25E-33
rs35978646	T	A	0.156716	0.198177	0.0266466	1.03E-13
rs142695953	A	C	0.1863	0.19257	0.0248973	1.04E-14
rs112266302	A	G	0.154145	0.15653	0.0268852	5.81E-09
rs62453594	C	T	0.122929	0.17067	0.0294441	6.78E-09
rs6967335	A	C	0.43172	-0.15636	0.0203828	1.70E-14
rs6957067	C	G	0.590692	-0.134484	0.0202349	3.01E-11
rs4731531	A	G	0.443021	0.113057	0.0201729	2.09E-08
rs7865719	G	A	0.55643	0.123757	0.0202607	1.01E-09
rs4263839	G	A	0.706524	0.12357	0.0223664	3.30E-08

rs10761659	G	A	0.527956	0.111962	0.0201122	2.59E-08
rs10748781	A	C	0.652344	-0.156268	0.0207885	5.60E-14
rs12769793	C	T	0.243859	0.132387	0.0229646	8.17E-09
rs7930763	A	G	0.439905	0.133479	0.0201251	3.30E-11
rs117115824	T	A	0.10226	-0.205664	0.0368118	2.31E-08
rs56086041	G	T	0.164664	-0.179266	0.0290559	6.84E-10
rs11658993	T	C	0.528238	0.135307	0.020139	1.83E-11
rs6017342	C	A	0.559104	0.149349	0.0203476	2.14E-13
rs6089926	T	C	0.207998	-0.162641	0.0257227	2.57E-10
rs4817986	T	G	0.247267	-0.157861	0.0238839	3.86E-11
rs9607629	G	A	0.125017	-0.203038	0.0320473	2.36E-10
rs9617090	T	C	0.372128	-0.163851	0.0210285	6.60E-15

T1DM, type 1 diabetes mellitus; SNP: single nucleotide polymorphism; beta: beta coefficient for effect allele; Se: standard error for effect allele; EA: effect allele; OA: other allele; EA: effect allele frequency.
